# Response to letter: Balancing risks and benefits of prucalopride for the treatment of chronic constipation in Asians

**DOI:** 10.1111/nmo.12033

**Published:** 2012-12-20

**Authors:** M Ke, D Zou, Y Yuan, Y Li, L Lin, J Hao, X Hou, H J Kim

**Affiliations:** * Clinical and Research Center of FGID & DGIM, Department of Gastroenterology, Peking Union Medical College HospitalBeijing, China; † Department of Gastroenterology, Changhai Hospital of ShanghaiShanghai, China; ‡ Department of Gastroenterology, Shanghai Ruijin HospitalShanghai, China; § Department of Gastroenterology, Qilu Hospital of Shangdong University, Capital Medical UniversityJinan, China; ¶ Department of Gastroenterology, Jiangsu Province HospitalNanjing, China; ** Department of Gastroenterology, Chaoyang Hospital, Capital Medical UniversityBeijing, China; †† Department of Gastroenterology, Union Hospital, Tongji Medical College, Huazhong Science Technology UniversityWuhan, China; ‡‡ Department of Internal Medicine, Kyung Hee University HospitalSeoul, Korea

We greatly appreciate the comments by Morais and Ford regarding balancing risks and benefits of prucalopride for the treatment in patients with chronic constipation in the Asia-Pacific region. The adverse events (AEs) have been presented as descriptive statistics in percentages of subjects reporting those individual events. Due to the difficulty in adjustment of the multiplicity issue in the large amount of safety data, it is common in regulatory filing that observed AE data are presented in percentages for each treatment group and discussed descriptively in the text.

From the forest plot shown by Morais and Ford, relative risk was calculated from percent of total AEs in prucalopride 2 mg and placebo groups in the first four individual studies.^1^–^4^ However, the relative risk in the Ke *et al.*^5^ study as calculated from total AEs should be 1.56 compared to 2.67 which was estimated from study drug-related adverse reactions.

The estimated relative risk was higher in patients from Asia-Pacific than in Caucasians. One of the possible explanations may be body weight which was lower in Asian-Pacific subjects (59.2 kg) than Caucasians (69.1 kg). The studies in Caucasians showed that most frequently reported treatment-emergent adverse events (TEAEs) in prucalopride group occurred mainly on the first day of treatment and the incidence of AEs excluding those on day 1 was similar to that observed in the placebo group.^1^–^3^ We analyzed the AEs by severity, timing, and duration of occurrences of individual common AEs. Our data were in line with those in Caucasian showing that majority of prucalopride-treated subjects reported these common TEAEs in the first week of treatment. They lasted for a few days (Table 1) with mild to moderate degree in severity.

**Table 1:** Duration of most frequently reported treatment-emergent adverse events (TEAEs) from the Asia-Pacific study*

TEAE duration (days)	Prucalopride 2 mg, *n* (%)	Placebo, *n* (%)
Diarrhea	169	72
1–2	146 (86.4)	69 (95.8)
3–5	15 (8.9)	3 (4.2)
>5	8 (4.7)	0
Headache	36	5
1–2	20 (55.6)	3 (60.0)
3–5	5 (13.9)	1 (20.0)
>5	11 (30.6)	1 (20.0)
Nausea	31	8
1–2	14 (45.2)	4 (50.0)
3–5	8 (25.8)	2 (25.0)
>5	9 (29.0)	2 (25.0)
Abdominal pain	18	8
1–2	11 (61.1)	7 (87.5)
3–5	1 (5.6)	0
>5	6 (33.3)	1 (12.5)

* *Ad hoc* analysis of prucalopride study in Asia-Pacific.

Further analysis of the serious adverse events (SAEs) demonstrated that fewer SAEs occurred in Asia-Pacific subjects compared to Caucasians in prucalopride and placebo (1.2% and 2.0% in Asia-Pacific *vs* 2.0%*vs* 2.7% in Caucasians [pooled data^1^–^3^], respectively). Moreover, a similar finding was observed for discontinuation of study treatment in prucalopride and placebo groups due to AEs (3.2% and 1.2% in Asia-Pacific *vs* 6.2% and 3.8% in Caucasians [pooled data^1^–^3^, respectively).

The conclusion as reported by Ke *et al.* confirms that prucalopride is safe and well tolerated by patients in the Asia-Pacific population. The most common AEs associated with prucalopride treatment were diarrhea, nausea, headache, and abdominal pain, most of which were mild to moderate in severity and transient, and spontaneously resolved in a few days.
